# Why should medical students (not) be recruited to care for patients with COVID-19?

**DOI:** 10.1186/s12909-020-02261-8

**Published:** 2020-10-02

**Authors:** Ivan Bank, Marjo Wijnen-Meijer

**Affiliations:** 1 Department of Transfusion Medicine, Sanquin Blood Bank, Amsterdam, The Netherlands; 2 Technical University of Munich, School of Medicine, TUM Medical Education Center, Ismaninger Straße 22, 81675 Munich, Germany

## Abstract

Worldwide it is being discussed whether medical students might be of help during the present COVID-19 epidemic. Although this question is probably a legitimate one, one should however discuss this thoroughly before deciding whether medical students are to be included in this kind of medical care on a larger scale.

Various arguments should be weighted, and potential tasks should be chosen carefully. This period could however be also an opportunity for medical students to learn things they would probably never learn about. Nevertheless, medical students have a deficit concerning knowledge about epidemics, and they are also not really well skilled in many hygiene measures. Furthermore, some of the known medical students’ behaviour could be a risk factor for further spread of the virus as well. Then, young adults are at risk of getting infected themselves. Last but not least, medical students in general are under a great deal of pressure from their studies which could lead to the development of anxiety and other mental disorders. One could only speculate on the effects of this epidemic on their further mental well-being. Therefore, medical students participating in direct care of patients with COVID-19 should first be trained well, and then properly supervised at all times. Only then it might be a really useful and exceptional experience, for healthcare, medical schools as well as for society.

## Background

Ever since the epidemic caused by COVID-19 has begun, governments are increasingly taking actions in order to try to slow down the spread of the infection, save as much as possible lives, and buy crucial time for either potential cure, healthcare capacity and/or production of a vaccine. Professionals involved in direct or indirect healthcare are doing everything within their bounds of possibilities to treat or facilitate patients. However, there is possibly coming a point in time when medical demand will outpace healthcare capacity, and that non-professionals will have to be included into healthcare tasks as well. Then, not only the army or retired medical professionals are or will be needed to be recruited for healthcare purposes, but for example also others like medical students.

This question was raised also by doctor Stokes, wondering if there is a plan in place for the involvement of senior medical students into emergency relief efforts [1]. Is this a feasible option? Time will tell. But, if we are to recruit medical students for healthcare purposes in time of crisis, which aspects should be taken into account? Let us try to list some issues concerning medical schools, medical students’ attitudes and behaviour during epidemics in general, as well as other students’ issues as known from earlier global outbreaks. This might help us to oversee the needs of medical students and possibly help institutions recognize what they should be aware of if they would like to let medical students participate in this particular medical care.

## The impact of COVID-19 on education

Most countries around the world are closing or they have already closed schools and educational institutions. Much of these actions were inspired by the epidemic with the severe acute respiratory syndrome (SARS) outbreak in 2003. It is not known whether this will also help stop the present pandemic [2]. By interrupting all human-to-human transmission, SARS itself was effectively eradicated.

Nowadays, if possible, online classes and courses are increasingly implemented. By doing so, risks of transmitting the virus within society are decreased, and in case of medical schools, transmission between clinic and other departments is possibly limited as well, minimizing the teaching burden on frontline providers. So, in most countries students are expected to engage in online learning activities and attendance continues to be recorded as usual. Students are strongly encouraged to make optimal use of their time to study using the online resources provided as these are based on core curriculum content.

It does not need to be explained that this epidemic may have a tremendous impact on student’s personal health as well as that of families, and that it may shift focus from studying to more essential matters. Then, all of these measures will delay programs and many medical students will have to deal with this, leading in some cases possibly not only to changing career opportunities, but perhaps also to financial problems. But how long will this last? Nobody knows how and when exactly the classes will be continued. Not only because of the fact that many teachers at medical schools are physicians, and they are now and possibly in the near future mostly needed in the clinical setting rather than giving classes. It is also to be expected that many hospitals and healthcare facilities, which are used to teach residents, trainees and clerks, will not be available for matters of education or regular curricula.

Finally, another major factor of importance for the education of medical students is the availability of protective equipment for students’ purposes, because they are becoming increasingly scarce, as it is even for essential personnel.

## Medical students’ behaviour during recent global threats

How did students or medical students act during earlier global epidemics?

Some of this may be illustrated by the pandemic influenza A/H1N1 2009 virus. That virus was especially virulent in young adolescents and pregnant women. Japanese students were studied. The virus occurred in almost 10% of these students [3]. It was found that attitudes and behaviors toward pandemic influenza control measures were different before and after the epidemic. As in this epidemic of COVID-19, the spread of influenza A/H1N1 2009 virus was also facilitated by social events, like club activities or social events, including student’s sports activities. The transmission was attenuated after temporary closure of such events and activities.

But, if we are interested in how medical students responded, this might be illustrated by an Australian study. During that same pandemic, perceptions and responses of students and university staff were studied by Van et al. [4]. Almost 80% of staff and students did not change lifestyle at all. Most respondents had not adopted any specific behaviour change, while 20.8% had started washing hands more often. Furthermore, students were more likely to attend classes and university buildings in comparison to staff.

Experiences concerning students’ hand hygiene and work with patients may be seen from the SARS outbreak in 2003. A Hong Kong study found that before the recognition of the SARS outbreak in 2003, about 35% of the students washed their hands before and 72.5% after they physically examined patients in the wards [5]. One year later, 60.3% washed hands before, and 100% after they had performed physical examination.

Another important tool concerns vaccination. How do students deal with vaccination in general?

From the influenza A/H1N1 pandemic, it is known that the vaccine was not accepted by all of the students contrary to most of the health care professionals that were offered the vaccine. For example, a Brazilian study amongst medical students showed a 91% adherence in the first year of availability in 2010 [6]. But, one year later, only 42% of the students took it. The investigators asked the study participants why they did not have taken it. Most students responded with “lack of time” and “have forgotten to take the vaccine”. And, clinical practice was also not a reason for these medical students to want the vaccine. The same concerns rise when vaccination is studied among staff from medical schools in France after the 2009 A/H1N1 influenza pandemic [7]. Only 18% were willing to be vaccinated, men significantly more than women (29% versus 9%), and professors/researchers more than administrative/technical staff (30% vs. 6%). Intention to be vaccinated was insufficient. If students are to be motivated to be vaccinated, efforts are needed for university staff too.

Finally, there also aspects concerning this issue that we cannot blame medical students at all because most of them just never had the opportunity to learn about risks and dangerous situations. Though, one may wonder how much medical students are taught about disaster medicine or these kind of issues at all. A survey among EU countries was performed by Ingrassia et al. about how much and what medical students learn about disasters [8]. Some universities teach this subject more or less, mostly in France, Germany and UK. There should however be more time for these issues and students were very motivated to learn these subjects [9]. Another survey among medical students showed that they are motivated to help during disaster situations or influenza pandemics, but also that they are not educated for disaster situations [10]. For example, the self-estimated capability to deal with an influenza pandemic was 3.92/10.

## Potential risks

There may come a point when the epidemic cannot be managed by regular systems and that non-professionals will need to be involved in medical care as well. Naturally, first of all those with some useful experiences or basic knowledge of medicine will be included. In general, during most of the known emergencies there is too little time and/or other means to help someone prepare for a new and difficult task. This will increase risk of making mistakes, spreading COVID-19 or getting ill. Next to medical students, also their families, peers and friends may potentially be at increasing risk. Contrary to former beliefs, adolescents and younger adults are also at risk of symptomatic COVID-19, as well as spreading this virus. Therefore, they should in general avoid as much as possible clinical settings, both professionally and privately [11].

If medical students are involved in care for patients with COVID-19, or they get tasks at departments where there is an increased risk of getting infected, they need to be provided the same scarce personal protective equipment (PPE) as medical professionals. At this moment that could be an important practical reason for not having students at the hospitals. The use of protective equipment is however another relevant issue. During crises with contagious agents, protective equipment is essential. Although medical students and trainees are taught during their rotations about how to use them, this is not always structural. This can be illustrated by a study from two hospitals in Cleveland, USA [12]. The students performed simulations of contaminated PPE removal. It appeared that only 41% received prior training, and that none of them had received training requiring demonstration of proficiency. Furthermore, 93% of the medical students had insufficient knowledge of how to wear the clothing and even 44% contaminated their own skin with fluorescent lotion.

Then, students need and should be provided sufficient supervision, at any time, especially during situations that they could neither have been trained nor taught about. During this epidemic potential supervisors have so many tasks that supervision can be missed while students wanting to show their skills or ambitions, are at some risk of doing harm to themselves, patients or colleagues.

These risks are perhaps not only hypothetical, because at the moment it is discussed whether it is possible to offer final year medical students early provisional registrations to help staffing levels during the covid-19 pandemic [13]. ‘As a fully registered doctor, trainees can practice with less supervision, including a wider range of prescribing responsibilities and, in some areas, the ability to independently discharge patients from hospital care.’

As stated before, medical students are not always vaccinated. In case that they are indeed working in health care, then there are not only risks of being infected with COVID-19, but also for example by other viruses, which especially at this moment may add diagnostically or treatment difficulties. A study from Minneapolis showed that only 23% of medical students being on clinical rounds were vaccinated against influenza, and that most of them do not even find it necessary for themselves as healthcare providers [14]. Although there are also many professionals not vaccinated, this may be taken for granted, but the same study also found that one third of the medical students had had needlestick exposure, and that an astonishing low 52% of them only told someone that that had happened.

Finally, medical students are known to have an increased risk of developing mental problems. It is known that students, especially medical students all over the world, appear to be frequently more anxious in general, with a prevalence rate of anxiety among medical students up to 33.8% as found in a large meta-analysis [15]. Furthermore, there is a growing number of observations about students’ problems and mental distress [16]. The various demands of medical education pose a challenge to personal well-being, sometimes even leading to burnout or, for example, erosion of empathy [17]. In the case of COVID-19 outbreak, lives of everyone including the lives of students are changed substantially. Then, students are not used to being isolated or to seeing so many ill people, and despite needs, even families cannot be always together to help them or encourage them if necessary. So, this situation has for certain a psychological impact, as did for example the epidemic of SARS earlier. This infection had caused anxiety of unprecedented proportion, and the occurrence of anxiety was common in medical students [18]. Anxiety levels, as well as perception of the crisis were investigated in them, about three weeks after the first reported SARS-related death in Malaysia. Both younger and more senior students were anxious, and showed sometimes fear. Students at the beginning of education however expressed significantly greater degree of anxiety compared to phase II students in relation to attendance and personal protection in hospital, and in meeting people coughing in public places. However, it could be that senior students were more mature and had more time spent in clinical wards giving them more tools to cope with the situation.

## Students’ matters

In various countries medical student support teams are helping to coordinate the new and unexpected situation. There are not only many questions about for example exams being delayed, which online training should be done, personal health or financial problems induced by the situation, but also questions about how to volunteer. So for example, medical students from University of Queensland, Australia, are only permitted to work in clinical settings ‘when adequate PPE of the type appropriate to the specific hazards is available and fitted and used correctly.’ The students are encouraged to ‘contact the local Clinical Unit for advice in case you have concerns about the availability of the correct PPE to conduct work safely in your placement location.’

In some countries like The Netherlands, medical students are getting involved in the pandemic through contact tracing as well as other useful tasks being possible by phone. For example, medical students are helping the Dutch Blood bank by calling potential donors who have experienced the infection themselves and are willing to donate convalescent plasma, a potential therapeutic option for patients with COVID-19 that needs to be studied more thoroughly. They are also increasingly getting involved in helping general practitioners by calling patients or during triage.

By allowing students to play an active role in such a crisis situation, this can contribute positively to the professional identity formation of medical students. Important factors here are the feeling that they can make a relevant contribution to patient care and be part of a team of healthcare professionals [19]. On the other hand, excluding students in such a situation can have a negative effect on their professional identity development.

Students themselves are organizing other ways of help, also possible useful, like the medical students from Singapore and Malaysia who started the #MoreViralThanTheVirus campaign. It is an initiative that urges youngsters to stay home to contain the COVID-19 pandemic. Another example of an initiative from students may be found on the internet [20].

Finally, this epidemic and medical students helping may not only be an opportunity for them to learn and pick up never seen or told experiences, but periods spent in healthcare helping during the COVID-19 epidemic might perhaps also be used by medical schools as substitution for particular clerkships in appropriate cases. However, this may never be the primary goal because many would find that to be unethical.

## Conclusion

At this moment, information, knowledge, attitudes, and practices towards COVID-19 are essential, independent of the fact if individuals are asked to come and help. With clear instructions and enormous efforts, China has so far shown to be able to change attitudes of many people and introduce new measures. Meanwhile, in most of the countries there will come a moment that everyone including volunteers and students will be needed to fight this epidemic, and support healthcare. Medical students, especially clerks could be of great help during this crisis, especially if the epidemic persists. If medical students are however structurally to be recruited, they should be instructed well what they have to do and how they have to do it. Supervision is inevitable. However, some common students’ characteristics and issues need to be addressed as was noted from previous epidemics.

Perhaps this opportunity might be a great one to start teaching medical students as soon as possible about for example management of outbreaks, existing protocols, or for example telehealth methods, so they could be sooner prepared or ready to help. If this additional classes are done by for example e-learning technology or organized internationally, perhaps the costs could be shared by more countries, and this could be an opportunity for medical schools to share more expertise and strengthen their present collaborations.

Inadequate attitudes about immunity to vaccine-preventable diseases as well as optimal hygiene measures need to be addressed. This should probably be also the case in medical university personnel, because they are also close link to patients, clinical doctors and students, so both medical students and staff should receive more information to increase vaccine coverage.

Despite all risks and current problems, if medical students are to be included in our global fight against COVID-19, let them help us by giving them the best possible tools and arms.

## Data Availability

Not applicable.

## Abbreviations

COVID-19: Coronavirus disease 2019
SARS: Severe acute respiratory syndrome
